# Descaling psoriasis narratives on TikTok: A cross‐sectional study

**DOI:** 10.1111/srt.13877

**Published:** 2024-07-22

**Authors:** Hamzah Ahmed

**Affiliations:** ^1^ University of Birmingham Birmingham UK

**Keywords:** Dermatology, Psoriasis, TikTok

Social media is increasingly being utilised by patients to obtain knowledge regarding their health conditions. TikTok, one of the biggest players in social media, has seen rapid growth within recent years and as such has become a major platform for seeking medical information. Therefore, it is important to ensure that the content being displayed is reliable and evidence based. This cross‐sectional study is based on the work by Oulee et al.^1^ “Atopic dermatitis on TikTok™: a cross‐sectional study”. Here, authors analysed videos on TikTok pertaining to atopic dermatitis. In our study, we will adopt a similar approach but instead analyse content on TikTok related to psoriasis.

To carry out our study, we first searched TikTok using “#Psoriasis” and navigated our way to the hashtag section. From here, we selected the first 200 videos to include in our study. On initial screening, 65 videos had to be excluded from the study due to them not being in English and 1 video had to be removed, as its content was based on seborrheic dermatitis rather than psoriasis. This process is outlined in Figure 1. Therefore, our final study size was 134 videos in total. The data points we collected are as follows. Firstly, the creator of each video was categorised into patient, dermatologist, health/wellness guru or other (hairdressers, tattoo artists, relatives of patients, etc.). Then, the content of each video was categorised into personal, educational or humorous. For each video, we also made a note of the number of likes it had received. Specifically for educational content, we used the NICE guidelines^2^ and NICE clinical knowledge summaries^3^ to determine whether the information being shared was reliable or not. The results of our study are:

**FIGURE 1:**
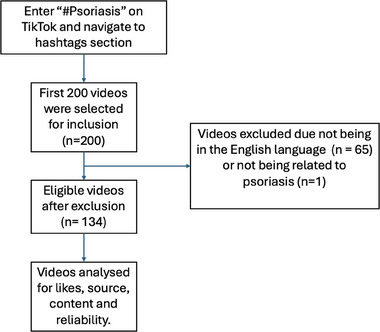
Flowchart showing which videos were included and excluded for analysis. (Adapted from Zheng et al.^4^).

Regarding creators of the videos, we found that 54% (*n* = 73) were produced by patients with psoriasis, 12% (*n* = 16) by dermatologists and 11% (*n* = 15) by health/wellness gurus. The remaining (*n* = 30) were made up of a combination of “other” individuals (previously discussed).

Content created by patients tended to be focused on what it is like living with psoriasis. Specifically, we found that 49% of these videos showed the physical removal of psoriatic plaques using a fine‐tooth comb. These were very popular on the platform, obtaining around 5 million likes in total. This trend is quite concerning given that it can prolong and even worsen psoriasis due to the Koebner phenomenon.^5^

Educational content on TikTok was primarily produced by either Dermatologists or Health/wellness gurus (Table 1). As mentioned, this content was evaluated to determine its reliability using the NICE guidelines^2^ and NICE Clinical Knowledge summaries.^3^ We found that of the 47 educational videos, around 55% (*n* = 26) were classed as reliable whilst the remaining 45% (*n* = 21) were deemed unreliable. Analysing the reliable content, the primary producers were dermatologists. Here, information such as the aetiology of psoriasis and its main treatment methods were shared. With regards to the unreliable content, the primary producers were health/wellness gurus. This information was mainly centred around the theme of discontinuing prescribed medication in favour of making dietary changes. This method was suggested to provide superior relief for individuals with psoriasis.

**TABLE 1:** Creators of reliable and unreliable educational videos.

Creator	Reliable videos	Unreliable videos
Dermatologist	16	0
Health/wellness Guru	2	13
Other	8	8

When looking at likes, our main analysis centred around educational content. We found that content we deemed reliable obtained a total of 2 million likes, whereas content deemed unreliable only obtained 850 000 likes. Whilst a positive revelation, it is still clear misinformation on TikTok gains considerable attention. In particular, one video we found in the study, obtaining 400 000 likes, stated that there was a causal link between psoriasis and a yeast found in the body! We also analysed the number of likes obtained by each source. Please see Figure 2 for more details.

**FIGURE 2:**
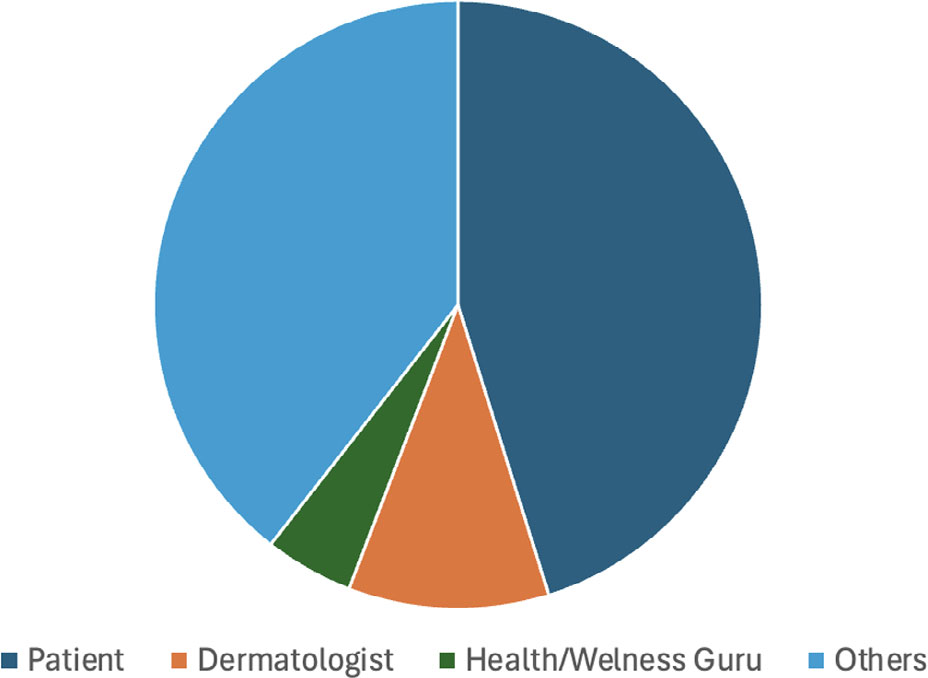
Proportion of likes gained by each source.

In conclusion, our study found that TikTok is filled with considerable amounts of misinformation. Firstly, we found that a significant quantitiy of patient‐generated content is centred around the removal of psoriatic plaques. If this is seen and emulated by others with scalp psoriasis, it could result in worsening and prolongation of the patient's condition. Secondly, TikTok is home to numerous health/wellness gurus, which produce large amounts of misleading content. To address these issues, we advise all healthcare professionals to caution patients when it comes to seeking health information on social media. They should be made aware that information is not always reliable and instead should be directed to more reputable sources. Another option is that healthcare professionals could increase their presence on social media. This will alert them to common themes of misinformation, and if they produce their own content, it increases the amount of evidence‐based material on the platform. It is our hope that the enactment of the aforementioned ameliorates the negative effects discussed.

## Data Availability

The data that support the findings of this study are available from the corresponding author upon reasonable request.
